# Polyamine-Mediated Transcriptional Regulation of Enzymatic Antioxidative Response to Excess Soil Moisture during Early Seedling Growth in Soybean

**DOI:** 10.3390/biology9080185

**Published:** 2020-07-22

**Authors:** Gagandip K. Sidhu, Pham Anh Tuan, Sylvie Renault, Fouad Daayf, Belay T. Ayele

**Affiliations:** 1 Department of Plant Science, 222 Agriculture Building, University of Manitoba, Winnipeg, MB R3T 2N2, Canada; gsidhu.4@gmail.com (G.K.S.); anh.pham@umanitoba.ca (P.A.T.); fouad.daayf@umanitoba.ca (F.D.); 2 Department of Biological Sciences, University of Manitoba, Winnipeg, MB R3T 2N2, Canada; sylvie.renault@umanitoba.ca

**Keywords:** cotyledon, enzyme activity, gene expression, root, shoot, spermidine, spermine

## Abstract

This study examined the expression patterns of antioxidative genes and the activity of the corresponding enzymes in the excess moisture-stressed seedlings of soybean in response to seed treatment with polyamines, spermine (Spm) and spermidine (Spd). At the 4 day after planting (DAP) stage, the excess moisture impaired the embryo axis growth, and this effect is associated with the downregulation of *superoxide dismutase* (*GmSOD1*) expression and SOD activity in the cotyledon. Seed treatment with Spm reversed the effects of excess moisture on embryo axis growth partly through enhancing glutathione reductase (GR) activity, in both the cotyledon and embryo axis, although no effect on the *GmGR* expression level was evident. Excess moisture inhibited the shoot and root growth in 7 DAP seedlings, and this is associated with decreased activities of GR in the shoot and SOD in the root. The effect of excess moisture on shoot and root growth was reversed by seed treatment with Spd, and this was mediated by the increased activities of ascorbate peroxidase (APX), catalase (CAT) and GR in the shoot, and APX in the root, however, only GR in the shoot appears to be regulated transcriptionally. Root growth was also reversed by seed treatment with Spm with no positive effect on gene expression and enzyme activity.

## 1. Introduction

Soybean is one of the most economically important crops cultivated around the world for food, oil and industrial purposes. However, its production is negatively impacted by several abiotic stress factors including excess moisture, which impedes gas diffusion and thereby causes a hypoxic growth environment [1]. Excess moisture-induced oxygen deprivation leads to the inhibition of cellular respiration for which oxygen is required as a terminal electron acceptor, and consequently disrupts all downstream cellular processes for which energy is required. It also triggers alterations in gene expression, protein synthesis and degradation, and other cellular metabolic activities [2]. All these factors negatively affect plant growth and developmental processes, and thereby cause substantial reduction in the yield of dryland crop species such as soybean, which are known to lack the ability to tolerate low oxygen growth conditions [2,3].

Germinating seeds and post-germinative seedlings of dryland crop species are very sensitive to excess moisture-induced injuries that negatively affect seedling establishment and the subsequent growth and developmental phases [4,5]. The process of seed germination begins with imbibition, which involves a rapid uptake of water by the dry seed and the activation of cellular respiration and other essential physiological pathways, and terminates with radicle protrusion through the seed-covering layers [6]. Subsequently, the coordinated mobilization of storage reserve and post-germination growth of the embryo axis proceeds until the seedling is established. The flooding of imbibing soybean seeds results in seed injury and a substantial decrease in germination percentage, the extent of which increases with the duration of flooding [4]. Excess soil moisture has also been reported to inhibit the growth of seedlings in other dryland crop species such as maize, and this has been shown to be accompanied by oxidative stress that leads to increased lipid peroxidation, membrane permeability and DNA damage [7]. These adverse effects of excess moisture on plant growth and development appear to be associated with changes in gene and protein expression patterns [8,9].

Oxidative stress is induced following the exposure of plants to abiotic stress factors, mainly due to the disruption of the balance between the accumulation of reactive oxygen species (ROS) such as superoxide radicals (O_2_∙^−^), hydrogen peroxide (H_2_O_2_), hydroxyl radicals (OH∙^−^), and the activity of ROS scavenging enzymatic and non-enzymatic antioxidative systems [10,11]. The enzymatic antioxidative system is an essential mechanism for fine-tuning the regulation of cellular ROS levels, which in turn influence the proper functioning of the ascorbate–glutathione cycle, an important non-enzymatic antioxidative system [10,12]. The first type of ROS produced in plants is generally O_2_∙^−^, a highly reactive molecule that is immediately converted to a less reactive form of ROS, H_2_O_2_, by the action of superoxide dismutase (SOD), an enzyme that functions as a first line of defense against ROS [13]. The resulting H_2_O_2_ is subsequently detoxified by ascorbate peroxidase (APX) and/or catalase (CAT) [14]. Glutathione reductase (GR) and APX play roles in replenishing the levels of ascorbic acid and glutathione, which are essential to the ascorbate–glutathione cycle involved in the neutralization of ROS [10]. Excess soil moisture conditions reportedly lead to increased oxidative damage via the decreased activity of antioxidative enzymes and the consequent ROS accumulation at both the seedling and vegetative stages, while increased antioxidative enzyme activity leads to the enhanced scavenging of excess ROS and thereby reducing oxidative damage and improving the tolerance to excess moisture [7,15]. Although genes encoding these antioxidative enzymes have been isolated from several plant species and studied for their roles in conferring protection against different stress factors [11], the molecular basis underlying the regulation of the enzymatic antioxidative system in response to excess moisture stress is poorly understood in soybean, especially during the early post-germinative seedling stage.

Polyamines (PAs) are ubiquitous polycationic amines that influence a wide range of plant growth and developmental processes and mediate stress responses [16]. Due to their polycationic nature, PAs can interact with and stabilize negatively charged cellular macromolecules such as DNA, RNA, chromatin and proteins [16,17,18]. Although the endogenous concentrations of these compounds vary with plant species, PAs are abundant in seedlings and the actively growing tissues of plants, and occur throughout various cellular organelles [19,20,21]. The tetramine spermine (Spm), triamine spermidine (Spd), which serves as a precursor for Spm, and the diamine putrescine (Put) that serves as a precursor for Spd, are the most common forms of polyamines in plants [16], and they can exist in free or conjugated forms as hydroxycinnamic amides [18,22]. However, PAs with more amine groups such as Spm and Spd have been suggested to be more effective in stabilizing key macromolecules and cellular membranes, and enhancing stress tolerance [23]. Consistently, the treatment of seedlings or adult plants of different species such as bean (*Phaseolus vulgaris*), ginseng (*Panax ginseng*) and pistachio (*Pistacia vera*) with Spd and/or Spm prior to exposures to different stress factors, such as acid rain and salinity, increases the expression of antioxidative genes and the activity of the corresponding enzymes, leading to reduced H_2_O_2_ levels and enhanced growth [24,25,26]. Furthermore, studies with maize seedlings and welsh onion plants have shown the roles of Spd and/or Spm treatments in enhancing the activities of antioxidative enzymes and thereby improving growth under excess moisture stress [7,15]. Although these studies have provided some insights into the role of PAs in enhancing the enzymatic antioxidative system and plant growth in response to a variety of abiotic stress factors, the molecular mechanisms underlying the role of these growth-regulating compounds in controlling the enzymatic antioxidative machinery and growth of soybean seedlings under excess moisture stress are largely unknown. To gain insights into this phenomenon, the present study investigated the spatiotemporal expression patterns of antioxidative genes and the activity of the corresponding enzymes, and the growth of excess moisture stressed the soybean seedlings in response to seed treatment with PAs, specifically Spd and Spm.

## 2. Materials and Methods

### 2.1. Plant Materials and Seed Treatment Assays

The mature dry seeds of soybean (*Glycine max* L.) were surface sterilized first with 70% ethanol for 1 min followed by 1.2% sodium hypochlorite for 20 min, and finally the seeds were rinsed with sterile water (five times). The sterilized seeds (20 per plate) were placed between two layers of Whatman #1 filter paper in 9 cm Petri plates, and then moistened with 10 mL of sterile water (control) or solutions of PAs (Spd (4 mM) or Spm (2 mM)) (Sigma-Aldrich, St. Louis, MO, USA). The plates were then incubated at room temperature (−22 °C) in darkness for 24 h before being transferred to pots. In order to examine the effect of PAs (Spd or Spm) on germination, soybean seeds were imbibed with water or solutions of PAs (20 seeds per plate per replicate; 5 replicates per treatment) as described above for 3 days, and the seeds were monitored every 12 h and scored as germinated when the radicle emerged beyond the seed coat. The concentrations of Spd or Spm used in this study were determined based on the results of our preliminary experiments that examined a range of Spd or Spm concentrations.

### 2.2. Excess Moisture-Stress Treatment and Tissue Collection

Seeds imbibed for 24 h with sterile water and Spd or Spm solution were transplanted into 3 L plastic pots containing a 2:1 (*v:v*) mixture of clay and sand soils as described previously [27]. The pots were then placed in a growth chamber with 22 °C/20 °C (day/night) temperature, ~50% humidity and 16/8 h photoperiod cycles with a light intensity of −38 W/m^2^. Half of the pots from each type of seed treatment was subjected to regular watering/moisture, while the other half to excess moisture treatments. The pots for excess moisture treatment were first placed in bigger pots filled with water for 24 h to saturate the soil completely with water followed by drainage for a period of 24 h prior to transplanting. After transplanting, the pots were placed back in bigger pots filled with water and the water level in the bigger pots was monitored regularly and topped up when necessary, in order to maintain the saturated soil conditions until tissue collection, which was performed at 4 and 7 days after planting (DAP) into the pots. The control pots were watered with a pre-determined volume of 200 mL on the day of transplanting, and then 150 mL every day until tissue collection. Five uniform seedlings (per pot per replicate, 3 replicates per treatment) were collected at 4 DAP (referred hereafter to as 4 DAP seedlings), and they were rinsed and dissected into cotyledon and embryo axis tissues while those collected at 7 DAP (referred hereafter to as 7 DAP seedlings) were dissected into cotyledon, shoot (hypocotyl plus any initiating leaflet) and root tissues. Growth parameters including the fresh weights (all tissues) and lengths (embryo axis, shoot and root) were measured immediately after harvest while the tissues used for other analyses were immediately frozen in liquid nitrogen and then stored at −80 °C until further use.

### 2.3. RNA Extraction and cDNA Synthesis

Total RNA samples were extracted from each seedling tissue as described previously [28]. To eliminate the genomic DNA contamination, the RNA samples were digested with DNase (DNA-free kit; Ambion, Austin, TX, USA). The quality of the RNA samples was verified by gel electrophoresis while the purity was determined using spectrophotometer. The RNA samples were then used to synthesize the cDNAs using a reverse transcription supermix (Bio-rad, Hercules, CA, USA) according to the manufacturer’s protocol. The resulting cDNA samples were diluted 20× before use for real time qPCR assays.

### 2.4. Primers

Primers for the target and reference genes were designed using Primer3 software (Table 1). The specificity of the primers was confirmed by blasting their sequence against the GenBank database and melting curve analysis while the efficiency of the qPCR reactions was determined using the Ct slope method as described previously [29]. The primers were designed based on previously reported nucleotide sequences of the respective soybean genes. The primers for *GmAPX*s were designed from the nucleotide sequence that is conserved between *GmAPX* (GenBank ID: NM_001250856) and *GmAPX* (GenBank ID: NM_001248658) as the two *GmAPX* genes exhibited a very high degree of sequence similarity. Likewise, the primers for *GmCAT*s were designed from a nucleotide sequence that is conserved among the *GmCAT* (GenBank ID: NM_001250627), *GmCAT* (GenBank ID: NM_001249045), *GmCAT* (GenBank ID: NM_001250642) and *GmCAT* (GenBank ID: NM_001253092) genes, as the four *GmCAT* genes exhibited a very high level of sequence similarity. Nucleotide sequences of *GmSOD1* (GenBank ID: NM_001248369) and *GmSOD3* (GenBank ID: NM_001255882) were used to design the primers for the respective genes. The primers for the *GmSODB*s were designed from the nucleotide sequence that is conserved between *GmSODB* (GenBank ID: NM_001251557) and *GmSODB2* (GenBank ID: NM_001250972) as the two *GmSODB* genes exhibited a very high degree of sequence similarity. Previous studies in other plant species such as Arabidopsis, maize and watermelon have also shown that the SOD enzyme is encoded by more than a gene that appears to form a small multigene family [11]. The *GmGR* (GenBank ID: NM_001251077) nucleotide sequences were used to the design primers for the respective gene. The primers for the reference gene were designed using the *Gmβ-actin1* (GenBank ID: NM_001289231) nucleotide sequence.

### 2.5. Real-Time qPCR Assays

Real-time qPCR assays were performed on a CFX96 Real-Time PCR detection system (Bio-rad) using SsoFast EvaGreen Supermix (Bio-rad) and qPCR reaction mixtures as described previously [29,30]. Briefly, a total qPCR reaction volume of 20 µL containing 10 µL of EvaGreen Supermix, 5 µL of 20× diluted cDNA, 1.2 µL of 5 µM forward primer (final concentration 300 nM), 1.2 µL of 5 µM reverse primer (final concentration 300 nM) and 2.6 µL of diethylpyrocarbonate-treated water was used. The samples were plated in duplicate in 96-well PCR plates and subjected to the following thermocycler conditions: DNA polymerase activation at 95 °C (5 min) followed by 40 cycles of denaturation at 95 °C (15 s), annealing at 60 °C (30 s), and extension at 72 °C (30 s). The relative transcript level of each gene was determined using the 2^−ΔΔCt^ method [31] and *Gmβ-actin1* as a reference gene.

### 2.6. Protein Extraction

The total protein extraction was performed as described previously [32]. Briefly, frozen tissues (100 mg) were ground into fine powder in liquid nitrogen using mortar and pestle. The fine powder was mixed with 1 mL of a 100 mM potassium phosphate extraction buffer (pH 7.0) containing 1% polyvinylpyrrolidone, 1 mM ethylenediaminetetraacetic acid disodium salt (EDTA-Na_2_) and 5 mM ascorbic acid (AsA) in a mortar and pestle pre-chilled at 4 °C. The mixture was then centrifuged at 19,000× *g* at 4 °C for 20 min.

### 2.7. Enzyme Activity Assays

The superoxide dismutase (EC 1.15.1.1) activity was determined as described previously [33] with minor modifications. Briefly, a 2 mL reaction mixture containing 50 mM potassium phosphate buffer (pH 7.8), 13 mM L-methionine, 75 μM nitroblue tetrazolium (NBT), 0.1 mM EDTA and 5 to 20 μL protein extract was prepared, and the reaction was initiated by adding 4 μL of 1 mM riboflavin with a final concentration of 2 μM, followed by the incubation of the mixture under direct light for 10 min. One unit of the SOD activity was defined as the amount of enzyme that caused the 50% inhibition in the reduction of NBT at 560 nm.

Ascorbate peroxidase (EC 1.11.1.11) activity was determined by monitoring the oxidation of AsA at 290 nm (extinction coefficient 2.8 mM^−1^ cm^−1^) as described previously [34], with slight modifications. Briefly, 2 mL of the reaction mixture containing 50 mM potassium phosphate buffer (pH 7.0), 1 mM EDTA (1 mM), 5 mM AsA and 100 μL protein extract was prepared, and the reaction was initiated by adding 1 mM of H_2_O_2_. One unit of APX activity was defined as the amount of enzyme required for the oxidation of 1 μmol of AsA per minute.

The catalase (EC 1.11.1.6) activity was assayed by monitoring the consumption of H_2_O_2_ (extinction coefficient 39.4 mM^−1^ cm^−1^) at 240 nm as described before [35]. The assay was performed with a 3 mL reaction mixture containing 50 mM potassium phosphate buffer (pH 7.0), 13 mM of H_2_O_2_ and 100 μL protein extract, and the degradation of 1 μmol of H_2_O_2_ per minute was defined as one unit of CAT activity.

Glutathione reductase (EC 1.6.4.2) activity was assayed by monitoring the oxidation of nicotinamide adenine dinucleotide phosphate hydrogen (NADPH) (extinction coefficient 6.22 mM^−1^ cm^−1^) at 340 nm according to the method described by Fryer et al. (1998) [36] with minor modifications. Briefly, 1.45 mL reaction mixture contained 100 mM potassium phosphate buffer (pH 7.0), 1 mM EDTA, and 0.2 mM NADPH, 100 μL of oxidized glutathione (GSSG, 5 mM) and 50 μL of the protein extract. One unit of GR activity was defined as the amount of enzyme required to oxidize 1 μmol of NADPH per minute.

### 2.8. Statistical Analysis

The statistical analysis of the significant differences between the samples was performed using the Student’s *t*-test at a probability of *p* ≤ 0.05. Comparisons were made between the samples derived from the seedlings grown under regular watering (RM) and excess moisture (EM) conditions, and between the seedlings grown under excess moisture (EM) and those grown under the same excess moisture condition, with or without seed treatment with spermidine (EM + Spd) or spermine (EM + Spm).

## 3. Results

### 3.1. Germination

**Figure 1:**
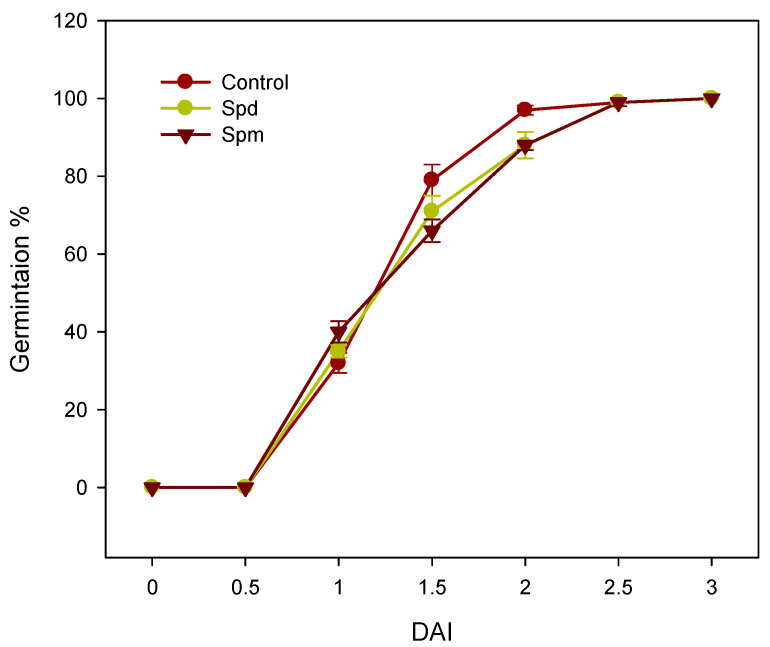
Germination percentage of the soybean seeds with or without the seed treatment with spermidine (Spd) or spermine (Spm). Data are the means ± SE, *n* = 5, where *n* refers to a batch of 20 seeds. DAI, days after imbibition.

The germination of the soybean seeds was monitored over a period of 3 days using radicle emergence as an indicator for the completion of germination. No marked effect of the seed treatment with Spd or Spm was observed on the germination of the soybean seeds except those seeds treated with Spd or Spm, which exhibited a slightly lower germination percentage at 1.5 and 2 days after imbibition (DAI) as compared to the PA-untreated control seeds (Figure 1).

### 3.2. Growth Response of Polyamine-Treated Seedlings to Excess Moisture

**Figure 2:**
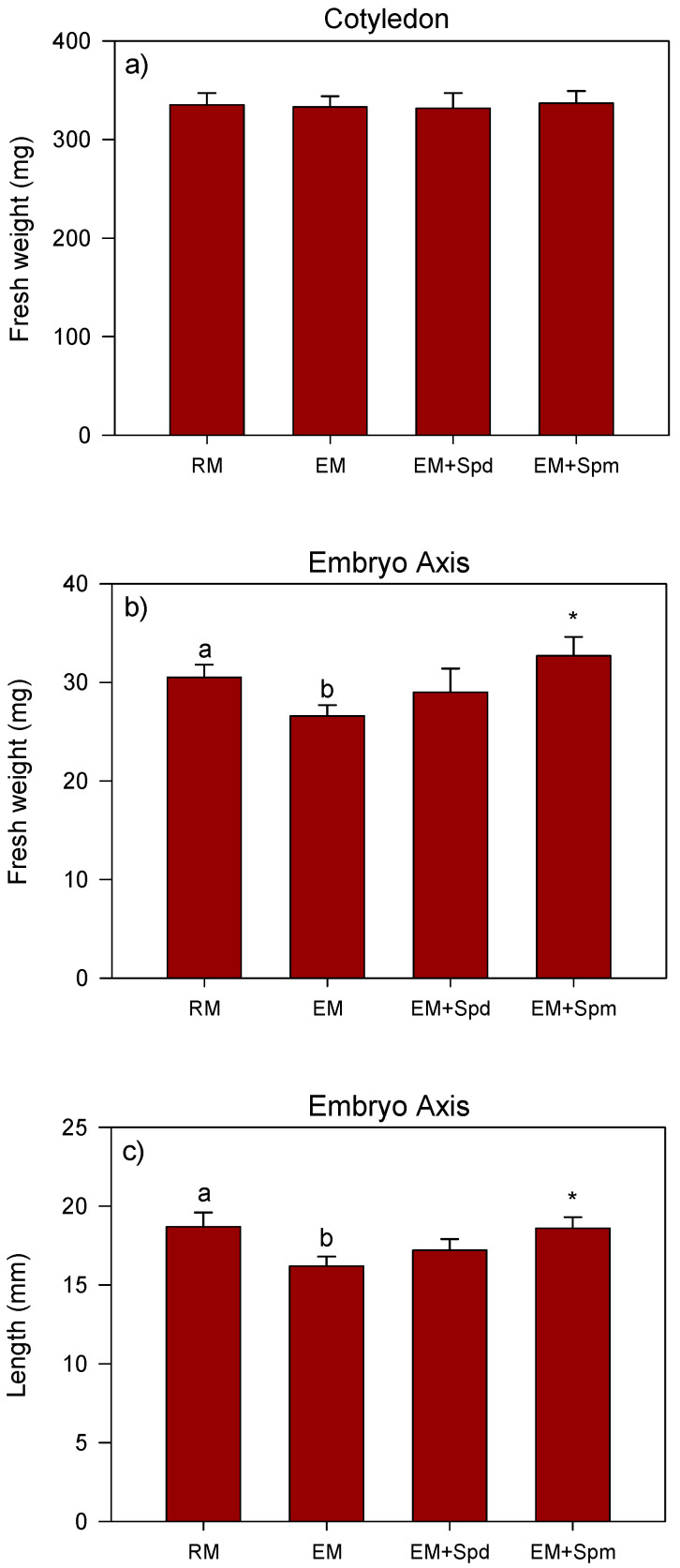
Fresh weight and length of the 4 DAP seedling tissues. Fresh weight (mg) of the cotyledon (**a**) and embryo axis (**b**), and length (mm) of the embryo axis (**c**) under regular watering (RM) and excess moisture (EM) conditions, with or without seed treatment with spermidine (EM + Spd) or spermine (EM + Spm). Data are the means ± SE, *n* = 12 to 15. Different letters indicate significant differences between the RM and EM while asterisks (*) indicate significant differences between the EM and EM + Spd or EM + Spm, *p* ≤ 0.05.

Excess soil moisture led to a reduction (~13%) of the embryo axis fresh weight and length in 4 DAP seedlings as compared to that observed in the control seedlings grown under regular watering conditions (Figure 2b,c). No effect of excess moisture was observed on the fresh weight of the cotyledon. While the seed treatment with Spm increased the fresh weight (23%) and length (15%) of the embryo axis of seedlings grown under excess moisture as compared to those grown under the same condition but with no seed treatment with the PAs, treatment with Spd did not have any effect. Seed treatment with either of the PAs did not affect the fresh weight of the cotyledons (Figure 2a).

**Figure 3:**
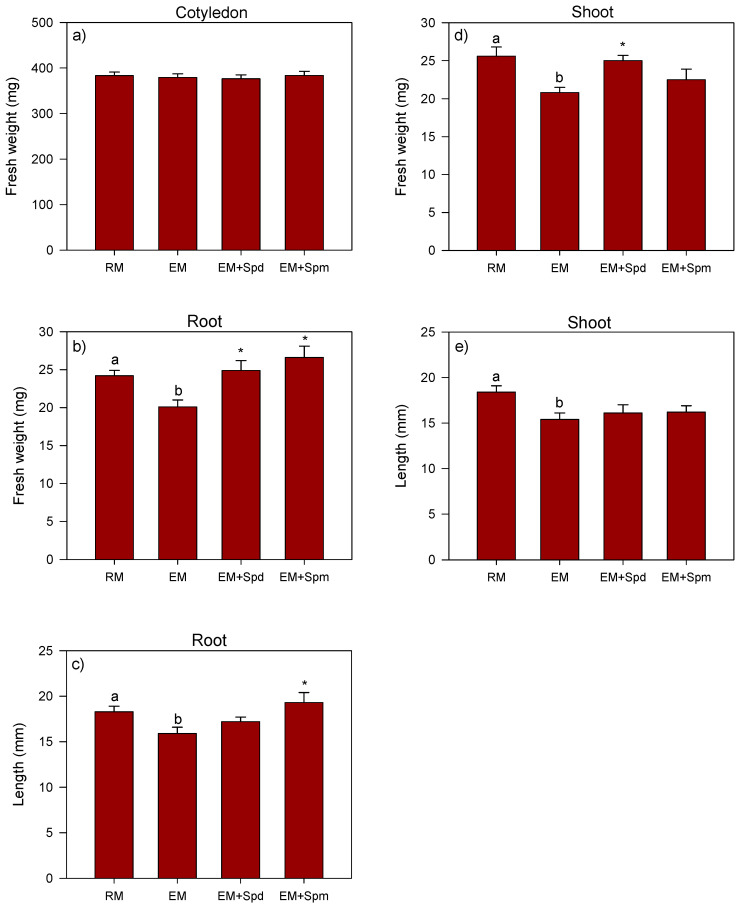
Fresh weight and length of the 7 DAP seedling tissues. Fresh weight (mg) of the cotyledon (**a**), root (**b**) and shoot (**d**), and length (mm) of the root (**c**) and shoot (**e**) under regular watering (RM) and excess moisture (EM) conditions, with or without seed treatment with spermidine (EM + Spd) or spermine (EM + Spm). Data are the means ± SE, *n* = 12 to 15. Different letters indicate significant differences between the RM and EM while asterisks (*) indicate significant differences between the EM and EM + Spd or EM + Spm, *p* ≤ 0.05.

The exposure of seedlings to excess moisture through the 7 DAP stage decreased the root (17%) and shoot (18%) fresh weights, as compared to that observed in the control seedlings grown under regular watering conditions with no effect on the cotyledon fresh weight (Figure 3a,b,d). It also decreased the root (12%) and shoot (16%) lengths (Figure 3c,e). Similar to that observed in 4 DAP seedlings, the seed treatment with Spd or Spm did not affect the fresh weight of the cotyledon of the seedlings grown under excess moisture. However, the treatment with Spd increased the root (23%) and shoot (20%) fresh weights with no effect on the shoot and root lengths of the seedlings grown under excess moisture as compared to those grown under the same conditions but with no seed treatment with the PAs, while the treatment with Spm increased both the root fresh weight (32%) and length (21%), with no apparent effect on the shoot fresh weight and shoot length.

### 3.3. Response of the Expression of Antioxidative Genes to Excess Moisture

#### 3.3.1. Ascorbate Peroxidase Genes

**Figure 4:**
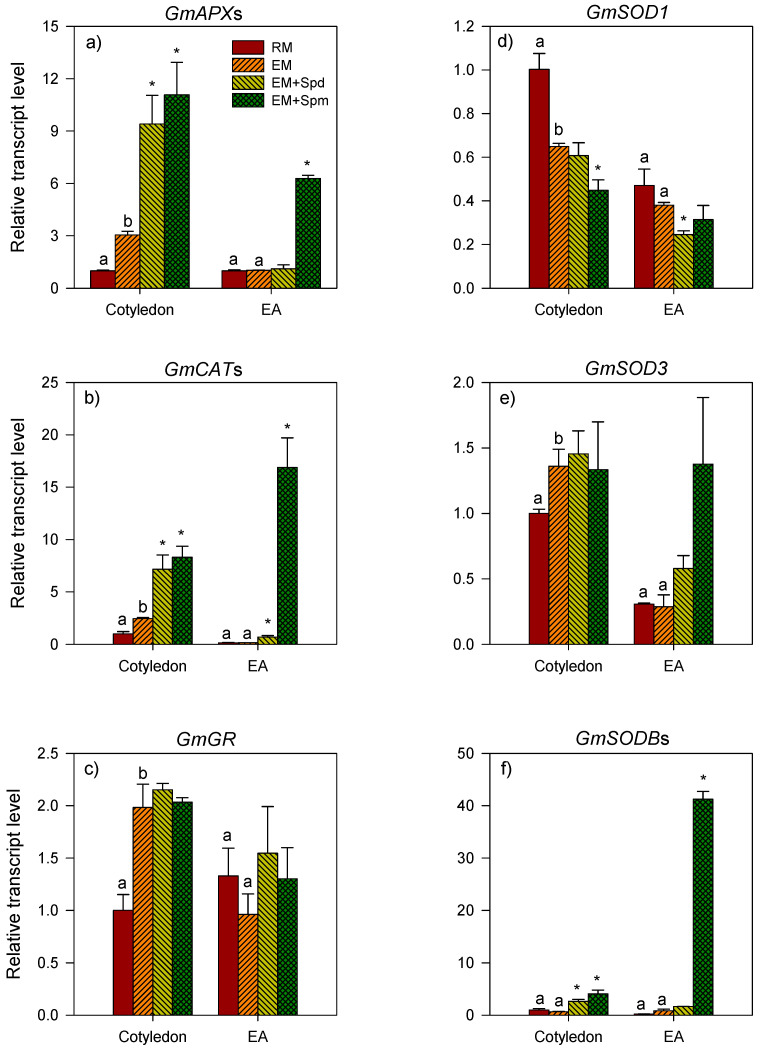
Expression of the genes encoding antioxidative enzymes in the tissues of 4 DAP seedlings. Relative transcript levels of *GmAPX*s (**a**), *GmCAT*s (**b**), *GmGR* (**c**) and the members of the *GmSOD* gene family, *GmSOD1* (**d**), *GmSOD3* (**e**) and *GmSODB*s (**f**) in the cotyledon and embryonic axis (EA) tissues of 4 DAP seedlings under regular watering (RM) and excess moisture (EM) conditions, with or without seed treatment with spermidine (EM + Spd) or spermine (EM + Spm). Transcript level of each gene was determined using *Gmβ-actin1* as the reference gene and is expressed as relative to the transcript level in the cotyledon tissues under RM, which was set to 1. Data are the means of 3 biological replicates ± SE. Different letters indicate significant differences in the transcript levels between the RM and EM while asterisks (*) indicate significant differences between the EM and EM + Spd or EM + Spm, *p* ≤ 0.05.

**Figure 5:**
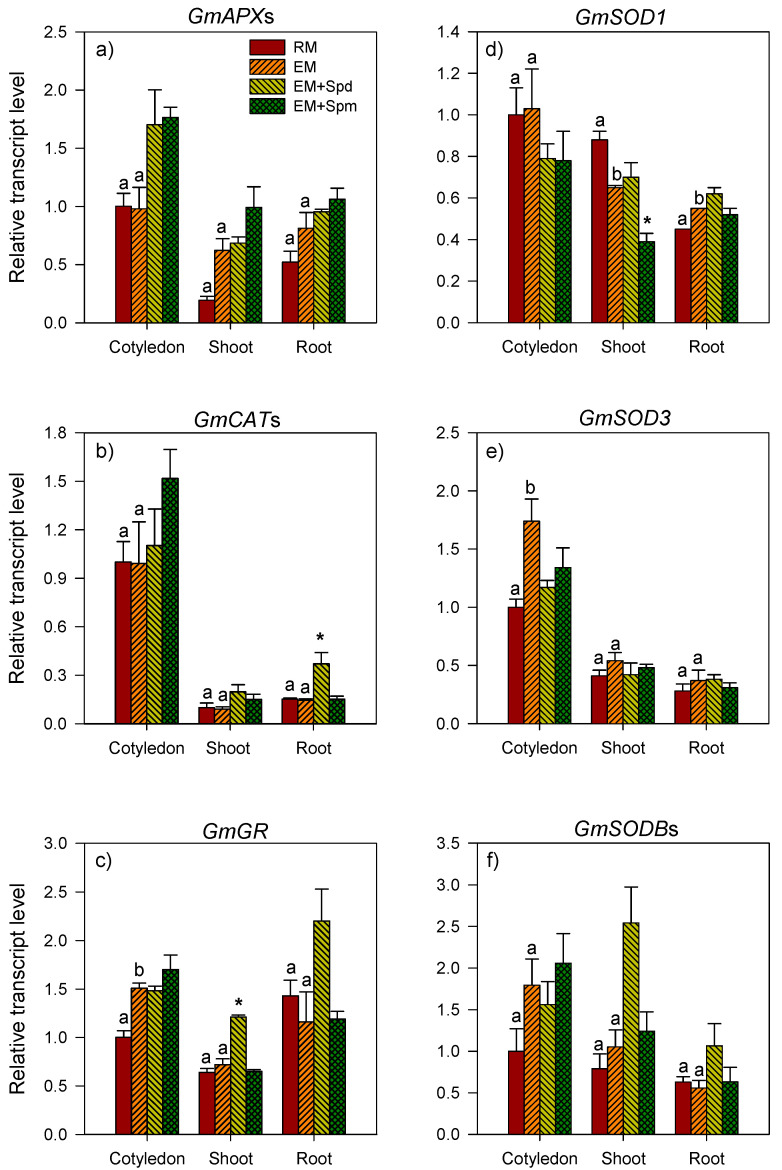
Expression of the genes encoding antioxidative enzymes in the tissues of 7 DAP seedlings. Relative transcript levels of *GmAPX*s (**a**), *GmCAT*s (**b**), *GmGR* (**c**) and the members of the *GmSOD* gene family, *GmSOD1* (**d**), *GmSOD3* (**e**) and *GmSODB*s (**f**) in the cotyledon, shoot and root tissues of seedlings under regular watering (RM) and excess moisture (EM) conditions, with or without seed treatment with spermidine (EM + Spd) or spermine (EM + Spm). Transcript level of each gene was determined using *Gmβ-actin1* as the reference gene and is expressed as relative to the transcript level in the cotyledon tissues under RM, which was set to 1. Data are the means of 3 biological replicates ± SE. Different letters indicate significant differences in the transcript levels between the RM and EM while asterisks (*) indicate significant difference between the EM and EM + Spd or EM + Spm, *p* ≤ 0.05.

The expression level of *GmAPX*s increased over 2-fold in the cotyledon tissues of excess moisture-stressed seedlings collected at 4 DAP with no significant change in its expression level in the embryo axis (Figure 4a). Relative to that observed in the control PA-untreated seedlings grown under excess moisture, the treatment of soybean seeds with Spd or Spm resulted in the enhanced expression level of *GmAPX*s (over 3-fold) in the cotyledon while only Spm caused an increased expression level (6.1-fold) of the same gene in the embryo axis (Figure 4a). Seed treatment with Spd did not affect the expression level of *GmAPX*s in the embryo axis. In the seedlings collected at 7 DAP, the level of *GmAPX*s expression in all the tissues was not affected by excess moisture (Figure 5a).

#### 3.3.2. Catalase Genes

The exposure to excess moisture led to an increase (2-fold) in the expression level of *GmCAT*s in the cotyledon of seedlings collected at 4 DAP while no significant change in its expression level occurred in the embryo axis (Figure 4b). However, the seed treatment with Spd or Spm enhanced the expression level of *GmCAT*s in the cotyledon (~3-fold or more) and embryo axis (~5-fold and 117-fold, respectively) of the excess moisture-stressed seedlings (Figure 4b). At the 7 DAP stage, there was no effect of excess moisture on the expression level of *GmCAT*s in all the seedling tissues including the cotyledon, root and shoot tissues (Figure 5b). Seed treatment with Spd led to an increase (over 2-fold) in the expression level of *GmCAT*s in the root but not in the other tissues of the seedlings exposed to excess soil moisture. The treatment with Spm did not affect the expression level of *GmCAT*s in all tissues (Figure 5b).

#### 3.3.3. Glutathione Reductase Genes

Similar to that observed for *GmAPX*s and *GmCAT*s, the expression level of *GmGR* increased in the cotyledon (~2-fold) of excess moisture-stressed seedlings collected at 4 DAP (Figure 4c). Excess moisture did not affect the expression level of the same gene in the embryo axis. The expression level of *GmGR* in both the cotyledon and embryo axis tissues of the seedlings exposed to excess soil moisture was not affected by the seed treatment with Spd or Spm (Figure 4c). Excess moisture also caused an increase (1.5-fold) in the expression level of *GmGR* in the cotyledons of the seedlings collected at 7 DAP with no apparent effect on its expression in the shoot and root tissues (Figure 5c). Relative to the corresponding PA-untreated/control seedlings, the Spd treatment increased (over 1.6-fold) the expression level of *GmGR* only in the shoot tissues of seedlings grown under excess moisture for 7 days (Figure 5c).

#### 3.3.4. Superoxide Dismutase Genes

With respect to the *GmSOD* gene family members (*GmSOD1*, *GmSOD3* and *GmSODB*s), the cotyledons of excess moisture-stressed seedlings collected at 4 DAP exhibited a decrease in the expression level of *GmSOD1* (1.5-fold) (Figure 4d), but an increase in the expression level of *GmSOD3* (1.5-fold) (Figure 4e). The expression level of *GmSODB*s remained unaffected (Figure 4d–f). Excess moisture did not affect the expression level of all the *GmSOD* genes in the embryo axis. Relative to the control PA-untreated seedlings, the seed treatment with Spm and Spd caused a decrease in the expression level of *GmSOD1* (1.5-fold or more) in the cotyledon and embryo axis, respectively, of the excess moisture-stressed 4 DAP seedlings (Figure 4d). Treatment with Spm or Spd on the other hand caused an increase in the expression level of *GmSODB*s in the cotyledon (6-fold) while only treatment with Spm resulted in an increase in the expression level of the same gene in the embryo axis (47-fold) of the seedlings exposed to excess moisture for 4 days (Figure 4f). The treatment seeds with Spm or Spd did not alter the expression level of *GmSOD3* in both the cotyledon and embryo axis tissues of the excess moisture-stressed seedlings collected at 4 DAP (Figure 4e).

At the 7 DAP stage, excess moisture enhanced the expression level of *GmSOD3* in the cotyledon and that of *GmSOD1* in the root, while repressing *GmSOD1* expression level in the shoot (Figure 5d,e). No effect of excess moisture was observed on the expression level of *GmSODB*s in any of the seedling tissues (Figure 5f). While the seed treatment with Spd or Spm did not affect the expression levels of *GmSOD3* and *GmSODB*s in any tissue of excess moisture-stressed 7 DAP seedlings as compared to that observed in the control PA-untreated seedlings, the treatment with Spd caused a decrease in the expression level of *GmSOD1* (1.7-fold) in the shoot tissue (Figure 5d).

### 3.4. Changes in the Activities of Antioxidative Enzymes in Seedling Tissues under Excess Moisture

**Figure 6:**
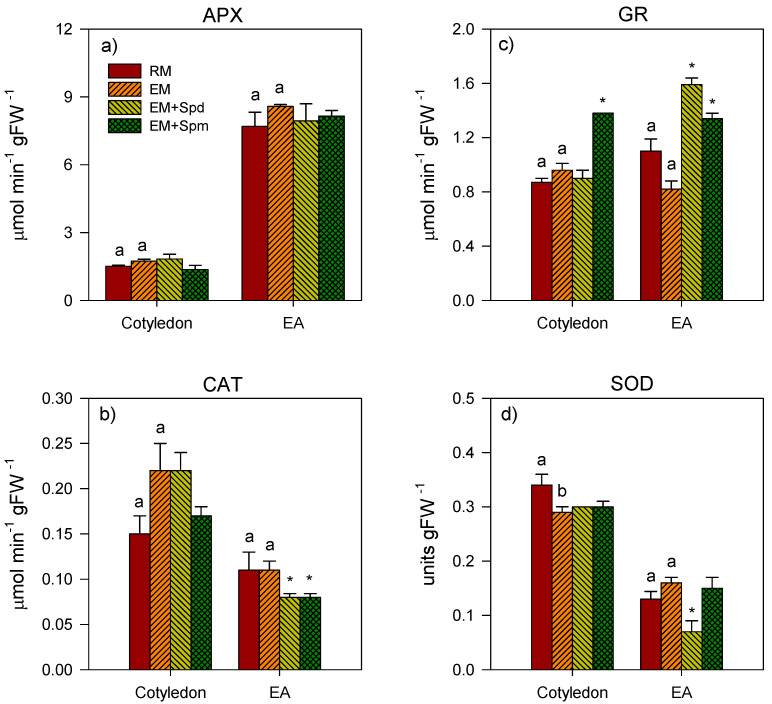
Antioxidative enzyme activity in the tissues of 4 DAP seedlings. Activities of ascorbate peroxidase (APX) (**a**), catalase (CAT) (**b**), glutathione reductase (GR) (**c**) and superoxide dismutase (SOD) (**d**) in the cotyledon and embryonic axis tissues of seedlings under regular watering (RM) and excess moisture (EM) conditions, with or without seed treatment with spermidine (EM + Spd) or spermine (EM + Spm). Data are the means ± SE, *n* = 2 to 3. Different letters indicate significant differences in enzyme activity between the RM and EM while asterisks (*) indicate significant difference between the EM and EM + Spd or EM + Spm, *p* ≤ 0.05.

**Figure 7:**
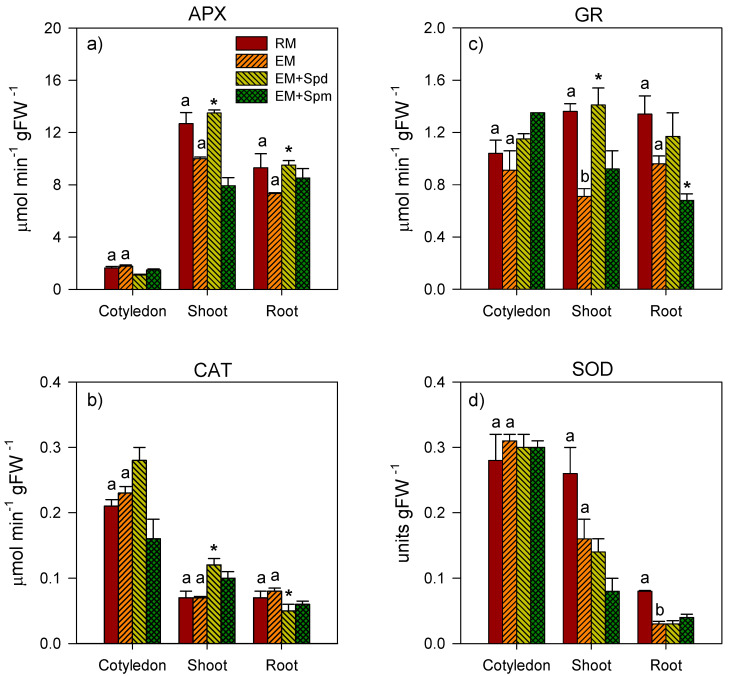
Antioxidative enzyme activity in the tissues of 7 DAP seedlings. Activities of ascorbate peroxidase (APX) (**a**), catalase (CAT) (**b**), glutathione reductase (GR) (**c**) and superoxide dismutase (SOD) (**d**) in the cotyledon, shoot and root tissues of seedlings under regular watering (RM) and excess moisture (EM) conditions, with or without seed treatment with spermidine (EM + Spd) or spermine (EM + Spm). Data are the means ± SE, *n* = 2 to 3. Different letters indicate significant differences in enzyme activity between the RM and EM while asterisks (*) indicate significant difference between the EM and EM + Spd or EM + Spm, *p* ≤ 0.05.

Excess moisture caused a 15% reduction of SOD activity in the cotyledon of 4 DAP seedlings (Figure 6), with no apparent effect on the activities of APX, CAT and GR in the same tissue. The exposure to excess moisture also did not affect the activities of APX, GR, SOD and CAT in the embryo axis of the seedlings collected at 4 DAP (Figure 6). Excess moisture treatment through the 7 DAP stage did not cause a significant alteration in the activities of APX, CAT, GR and SOD in the cotyledon; however, it caused 40% and 69% decreases in the GR activity of the shoot and SOD activity of the root, respectively (Figure 7).

### 3.5. Polyamine-Induced Modulation of Antioxidative Enzyme Activities under Excess Moisture

Seed treatment with Spm increased (44%) the activity of GR in the cotyledon of excess moisture-stressed seedlings collected at 4 DAP (Figure 6). Treatment with Spd, on the other hand, did not have any effect on the activities of all enzymes in the cotyledon. In the embryo axis, the treatment with Spd or Spm increased the GR activity (63% and 94%, respectively) under excess moisture while reducing the activity of CAT (27%). Spd treatment also caused a ~56% reduction in the activity of SOD.

With respect to the seedlings exposed to excess moisture through the 7 DAP stage, the seed treatment with Spd resulted in an increase in the activity of APX in both the shoot (35%) and root (30%) tissues (Figure 7). Furthermore, the Spd increased the activities of CAT (71%) and GR (99%) in the shoot tissue of excess moisture-stressed 7 DAP seedlings (Figure 7). In contrast, the seed treatment with Spd decreased the activity of CAT (38%) in the root tissues of excess moisture-stressed seedlings while treatment with Spm reduced the activity of GR (29%) in the same tissue.

## 4. Discussion

Seed germination and the seedling establishment are complex physiological processes regulated by a variety of environmental factors and endogenous signaling molecules such as polyamines. Seed treatment with PAs such as Spd has been shown to improve the seed germination percentage in crop species such as maize [37], while in other species such as white clover, Spd treatment was observed to improve the mean germination time [38]. However, the germination of soybean seeds was only slightly or not affected by the treatment with Spd or Spm. Given that the endogenous levels of PAs have been reported to vary with plant species, tissue types and developmental stages [21], our data might suggest that the mature seeds of soybean contain PAs at a level that is sufficient to support the germination process. Consistent with this hypothesis, the accumulation of putrescine, Spd and Spm has been reported in the mature and germinating seeds of some species such as bean and pea [39,40].

Previous studies have shown that excess moisture creates hypoxic conditions and thereby inhibit seedling growth in dryland crop species such as soybean and maize [4,7], and these effects of excess moisture are shown to be mediated by lipid peroxidation-induced oxidative stress and cellular damage [9,41]. In agreement with the reports of such studies, excess moisture-stressed soybean seedlings exhibited the reduction in embryo axis, shoot and root growth. Polyamines have been reported to alleviate the deleterious effects of abiotic stress factors on plant growth and development [42]. For example, Spd and Spm improve the root and shoot growth of seedlings in different plant species such as pistachio, maize and mung bean under a variety of stress factors including excess moisture, salt, high temperature and drought [7,25,43]. Consistent with these observations, the seed treatment with Spm improved the embryo axis and root growth of the seedlings grown under excess moisture for 4 and 7 days, respectively, while the treatment with Spd was able to reverse the negative effect of excess moisture on the shoot and root fresh weights of seedlings at 7 DAP. Given that the Spm and Spd are positively charged, the effects of these two PAs in alleviating the excess moisture-induced inhibition of the seedling tissues can be attributed to their ability to bind and stabilize negatively charged macromolecules and cellular membranes [15].

The disruption of the enzymatic antioxidative system, which leads to the excessive accumulation of ROS and thereby cellular oxidative damage, is one of the mechanisms by which excess moisture severely impairs plant growth and development [44]. It has been shown in crop species such as pea and mung bean that the mobilization of storage reserves from the cotyledon to the growing embryo axis of seedlings is associated with an increased rate of respiration and thereby the production of ROS, which play a critical role in cell elongation and signaling [45,46,47]. However, the exposure of seedlings to excess moisture-induced hypoxia can further increase in ROS accumulation beyond the scavenging capacity of the enzymatic and non-enzymatic systems, leading to oxidative stress [44,48]. Our data showed that the excess moisture-mediated reduction of embryo axis growth in 4 DAP seedlings is associated with decreases in the expression level of *GmSOD1* and the activity of the corresponding enzyme in the cotyledon, which serves as a reserve storage organ in soybean seeds. Furthermore, excess moisture led to the repression of GR activity in the shoot and SOD activity in the root tissues of 7 DAP seedlings. Owing to their first line of defense against ROS-induced injuries, the reductions in the activities of SOD and GR [11] likely contribute to the inhibition of shoot and root growth in the seedlings exposed to excess moisture. Given that GR is also an essential part of the ascorbate–glutathione pathway that generates antioxidant metabolites [11], the reduction of its activity in the shoot might also imply a decrease in the non-enzymatic antioxidant system. It is therefore necessary to consider testing this hypothesis in future studies. Consistent with our results, waterlogging has been shown to inhibit seedling growth in maize through altering the activities of antioxidant enzymes and enhancing the accumulation of ROS [7,26,43]. The prevalence of excess moisture stress-induced changes in the expression patterns of antioxidative genes and/or activity of the corresponding enzymes in the different tissues of soybean seedlings highlights the significance of the tissue-specific transcriptional regulation of antioxidative genes in controlling enzymatic ROS scavenging capacity [11,44].

Polyamines have been implicated in improving plant performance under abiotic stress conditions via stabilizing key macromolecules such as DNA/RNA and membrane lipids, and enzymes that are susceptible to ROS-induced oxidative damage [23,49]. They are also known to influence the levels of other metabolites that are involved in plant stress response, signaling and non-enzymatic antioxidation [22,49,50]. The treatment of soybean seeds with Spm enhanced the embryo axis growth in excess moisture-stressed 4 DAP seedlings, and this effect was associated with the transcriptional induction of the *GmAPX*, *GmCAT* and *GmSODB* genes in the embryo axis and the enhanced GR activity in both cotyledon and embryo axis tissues. Furthermore, the Spd treatment improved the shoot growth in excess moisture-stressed 7 DAP seedlings, and this effect of Spd was associated with the increased expression level of *GmGR* and the activities of APX, CAT and GR while its effect on enhancing root growth was associated with increased APX activity. Similarly, Spd treatment has been reported to enhance antioxidative enzyme activity and thereby reduce ROS levels and improve the growth of maize seedlings under excess moisture conditions [7]. Furthermore, exogenous PA treatment has been shown to improve growth in different plants species under a variety of abiotic stress factors, through influencing the expression levels of antioxidative genes and the activity of the corresponding enzymes [15,49,51]. In this study, substantial induction in the expression levels of the *GmAPX*, *GmCAT* and *GmSODB* genes in response to Spm treatment was observed in the embryo axis of excess moisture-stressed 4 DAP seedlings. The Spm treatment led to a 117-fold increase in the expression level of *GmCAT*s and a 47-fold increase in that of *GmSODBs,* while causing a much smaller increase (6.1-fold) in the expression level of *GmAPX*s. Despite the increases observed in the expression levels of these three genes in response to spm treatment, no increases were evident in the activities of the corresponding enzymes. In addition, our data showed increased GR activity in the cotyledon and embryo axis of excess moisture-stressed 4 DAP seedlings in response to the Spm treatment or to that of CAT and APX in the root and/or shoot of excess moisture-stressed 7 DAP seedlings in response to the Spd treatment despite the absence of any positive effect of the treatments on the expression levels of the corresponding genes. These results suggest that the expression of antioxidative genes is subjected to post-transcriptional regulation by PAs under abiotic stress conditions. In agreement with this, the post-transcriptional regulation of antioxidative genes has been reported to occur in other plant species under different stress factors, for example, in rice under salt stress and *Medicago truncatula* under drought stress [52]. However, we cannot exclude the possibility that the Spm/Spd-induced increases in the activity of GR, APX and CAT are mediated by hte transcriptional regulation of specific members of the respective gene families that are yet to be identified.

Of the three PAs found in plants, Spd and Spm were shown to have a positive effect on the metabolic and physiological responses of plants to stress factors as compared to the diamine Put [23,51,53]. However, Spd and Spm do not appear to affect the antioxidative enzyme response of plants, and thereby their performance under abiotic stresses, in the same way [15,50]. Our data showed that the Spm treatment enhances the embryo axis growth in excess moisture-stressed 4 DAP seedlings through increased GR activity, while the seed treatment with Spd did not improve the embryo axis growth despite the enhanced GR activity observed in the same tissue, suggesting the effects of Spm and Spd-induced antioxidative responses on plant growth under stress conditions are mediated by different mechanisms. At the later seedling growth stage, treatment with Spm led to improved root growth in excess moisture-stressed seedlings with no apparent induction in the expression levels of antioxidative genes or activity of the corresponding enzymes, suggesting the protective role of Spm against abiotic stress factors might involve non-antioxidative mechanisms. Consistent with this hypothesis, Spm, which is characterized by its longer chain and greater number of positive charges, has also been reported to have a more protective effect against abiotic stress factors such as acid rain than Spd, and this effect is likely attributed to its better membrane stabilizing properties [24]. Previous studies have shown the involvement of polyamine oxidase (PAO), which catabolizes PAs to maintain their homeostasis, in mediating plant responses to abiotic stress factors, for example, an increase in PAO activity has been reported in response to salt and hypoxic stress conditions [54,55]. Our data showed that the treatment with Spm did not improve the shoot growth in excess moisture-stressed 7 DAP seedlings. This along with the absence of Spm-mediated transcriptional activation of antioxidative genes or activity of the corresponding enzymes in the same tissue might suggest a decrease in the Spm level by the excess moisture-induced activity of PAO, which can convert Spm to Spd or oxidize it to H_2_O_2_ and a nitrogen-based compound [56]. In agreement with this hypothesis, the presence of the higher activity of PAO in the shoot and root tissues of soybean seedlings as compared to the cotyledon have been shown to be associated with a lower level of endogenous Spm than that of Spd [20].

In conclusion, our results highlight that excess moisture represses the embryo axis or root and shoot growth of soybean seedlings; however, this effect of excess moisture can be alleviated by seed treatments with Spm or Spd, which appear to enhance the enzymatic antioxidant system via the transcriptional or post-transcriptional regulations of specific antioxidative genes. However, the effects of Spm or Spd on the expression levels of antioxidative genes and/or activity of the corresponding enzymes and thereby growth appeared to vary with seedling tissue types and the stage/duration of their exposure to excess moisture stress. Although the results of study provide insights into the role of Spm or Spd in improving the root and shoot growth of soybean seedlings under excess moisture stress through the alteration of the enzymatic antioxidant system, further biochemical and genetic studies are required to elucidate the definitive roles of PAs in protecting soybean plants from excess moisture-induced oxidative damages.

## Figures and Tables

**Table 1:** Sequences of the primers used for the gene expression analysis.

Gene	Type	Sequence (5′ to 3′)	Amplicon Size	Accession Number
*Gmβ-actin1*	F ^a^	CGGTGGTTCTATCTTGGCATC	142	NM_001289231
	R ^b^	GTCTTTCGCTTCAATAACCCTA		
*GmAPX*s	F	GATGCGCTCCTCTAATGCTC	198	NM_001250856,
	R	AGAAATCGGCGTAGCTCAAA		NM_001248658
*GmCAT*s	F	CGCCTTCAATTCTCCCTTCT	99	NM_001250627,
	R	TCCAGCAGAATTGGACCTCT		NM_001249045, NM_001250642, NM_001253092
*GmGR*	F	GCGAGCTTCCTTTCTCCACT	92	NM_001251077
	R	CAGCAACTTCTTCGGCACAC		
*GmSOD1*	F	TGAAGGCTGTGGCAGTTCTT	83	NM_001248369
	R	GGTGGTTGGACCATTTCCCT		
*GmSOD3*	F	AATGGGACCACCCATGTGAC	106	NM_001255882
	R	CAGTGGAGTTGCAGCCATTG		
*GmSODB*s	F	CTGCTGCTGCAACACAATTT	141	NM_001251557,
	R	TCACAGCATTGGGACTCTTG		NM_001250972

^a^ F = forward primer; ^b^ R = reverse primer.

## Abbreviations

The following abbreviations are used in this manuscript:

APX	ascorbate peroxidase
CAT	catalase
DAI	days after imbibition
DAP	days after planting
EA	embryo axis
EM	excess moisture
GR	glutathione reductase
RM	regular watering
ROS	reactive oxygen species
SOD	superoxide dismutase
Spd	spermidine
Spm	spermine

## References

[1] Bailey-Serres J., Colmer T.D. (2014). Plant tolerance of flooding stress—Recent advances. Plant Cell Environ..

[2] Bailey-Serres J., Voesenek L.A.C.J. (2008). Flooding stress: Acclimations and genetic diversity. Annu. Rev. Plant Biol..

[3] Dat J.F., Capelli N., Folzer H., Bourgeade P., Badot P.-M. (2004). Sensing and signalling during plant flooding. Plant Physiol. Biochem..

[4] Wuebker E.F., Mullen R.E., Koehler K. (2001). Flooding and temperature effects on soybean germination. Crop Sci..

[5] Lee K.-W., Chen P.W., Yu S.-M. (2014). Metabolic adaptation to sugar/O_2_ deficiency for anaerobic germination and seedling growth in rice. Plant Cell Environ..

[6] Bewley J.D. (1997). Seed germination and dormancy. Plant Cell.

[7] Liu M.Y., Sun J., Wang K.Y., Liu D., Li Z.Y., Zhang J. (2014). Spermidine enhances waterlogging tolerance via regulation of antioxidant defence, heat shock protein expression and plasma membrane H+-ATPase activity in *Zea mays*. J. Agron. Crop Sci..

[8] Bailey-Serres J., Lee S.C., Brinton E. (2012). Waterproofing crops: Effective flooding survival strategies. Plant Physiol..

[9] Hashiguchi A., Sakata K., Komatsu S. (2009). Proteome analysis of early-stage soybean seedlings under flooding stress. J. Proteome Res..

[10] Apel K., Hirt H. (2004). Reactive oxygen species: Metabolism, oxidative stress, and signal transduction. Annu. Rev. Plant Biol..

[11] Gill S.S., Tuteja N. (2010). Reactive oxygen species and antioxidant machinery in abiotic stress tolerance in crop plants. Plant Physiol. Biochem..

[12] Bailly C., El-Maarouf-Bouteau H., Corbineau F. (2008). From intracellular signaling networks to cell death: The dual role of reactive oxygen species in seed physiology. C. R. Biol..

[13] Alscher R.G., Erturk N., Heath L.S. (2002). Role of superoxide dismutases (SODs) in controlling oxidative stress in plants. J. Exp. Bot..

[14] Ahmad P., Jaleel C.A., Salem M.A., Nabi G., Sharma S. (2010). Roles of enzymatic and nonenzymatic antioxidants in plants during abiotic stress. Crit. Rev. Biotechnol..

[15] Yiu J.-C., Liu C.-W., Yi-Tan Fang D., Lai Y.-S. (2009). Waterlogging tolerance of Welsh onion (*Allium fistulosum* L.) enhanced by exogenous spermidine and spermine. Plant Physiol. Biochem..

[16] Kusano T., Berberich T., Tateda C., Takahashi Y. (2008). Polyamines: Essential factors for growth and survival. Planta.

[17] Alcázar R., Altabella T., Marco F., Bortolotti C., Reymond M., Koncz C., Carrasco P., Tiburcio A.F. (2010). Polyamines: Molecules with regulatory functions in plant abiotic stress tolerance. Planta.

[18] Tiburcio A.F., Altabella T., Bitrián M., Alcázar R. (2014). The roles of polyamines during the lifespan of plants: From development to stress. Planta.

[19] Hussain S.S., Ali M., Ahmad M., Siddique K.H.M. (2011). Polyamines: Natural and engineered abiotic and biotic stress tolerance in plants. Biotechnol. Adv..

[20] Scoccianti V., Torrigiani P., Bagni N. (1990). Distribution of diamine oxidase activity and polyamine pattern in bean and soybean seedlings at different stages of germination. Physiol. Plant..

[21] Jiménez-Bremont J.F., Marina M., Guerrero-González M.d.l.L., Rossi F.R., Sánchez-Rangel D., Rodríguez-Kessler M., Ruiz O.A., Gárriz A. (2014). Physiological and molecular implications of plant polyamine metabolism during biotic interactions. Front. Plant Sci..

[22] Gupta K., Dey A., Gupta B. (2013). Plant polyamines in abiotic stress responses. Acta Physiol. Plant..

[23] Bouchereau A., Aziz A., Larher F., Martin-Tanguy J. (1999). Polyamines and environmental challenges: Recent development. Plant Sci..

[24] Velikova V., Yordanov I., Edreva A. (2000). Oxidative stress and some antioxidant systems in acid rain-treated bean plants: Protective role of exogenous polyamines. Plant Sci..

[25] Kamiab F., Talaie A., Khezri M., Javanshah A. (2014). Exogenous application of free polyamines enhance salt tolerance of pistachio (*Pistacia vera* L.) seedlings. Plant Growth Regul..

[26] Parvin S., Lee O.R., Sathiyaraj G., Khorolragchaa A., Kim Y.-J., Yang D.-C. (2014). Spermidine alleviates the growth of saline-stressed ginseng seedlings through antioxidative defense system. Gene.

[27] Nguyen T.-N., Tuan P.A., Mukherjee S., Son S., Ayele B.T. (2018). Hormonal regulation in adventitious roots and during their emergence under waterlogged conditions in wheat. J. Exp. Bot..

[28] Mukherjee S., Liu A., Deol K.K., Kulichikhin K., Stasolla C., Brûlé-Babel A., Ayele B.T. (2015). Transcriptional coordination and abscisic acid mediated regulation of sucrose transport and sucrose-to-starch metabolism related genes during grain filling in wheat (*Triticum aestivum* L.). Plant Sci..

[29] Yao Z., Liu L., Gao F., Rampitsch C., Reinecke D.M., Ozga J.A., Ayele B.T. (2012). Developmental and seed aging mediated regulation of antioxidative genes and differential expression of proteins during pre- and post-germinative phases in pea. J. Plant Physiol..

[30] Nguyen T.-N., Son S., Jordan M.C., Levin D.B., Ayele B.T. (2016). Lignin biosynthesis in wheat (*Triticum aestivum* L.): Its response to waterlogging and association with hormonal levels. BMC Plant Biol..

[31] Livak K.J., Schmittgen T.D. (2001). Analysis of relative gene expression data using Real-time quantitative PCR and the 2−ΔΔCT method. Methods.

[32] Agarwal S., Sairam R.K., Srivastava G.C., Tyagi A., Meena R.C. (2005). Role of ABA, salicylic acid, calcium and hydrogen peroxide on antioxidant enzymes induction in wheat seedlings. Plant Sci..

[33] Dionisio-Sese M.L., Tobita S. (1998). Antioxidant responses of rice seedlings to salinity stress. Plant Sci..

[34] Nakano Y., Asada K. (1981). Hydrogen peroxide is scavenged by ascorbate-specific peroxidase in spinach chloroplasts. Plant Cell Physiol..

[35] Aebi H. (1984). Catalase in vitro. Methods Enzymol..

[36] Fryer M.J., Andrews J.R., Oxborough K., Blowers D.A., Baker N.R. (1998). Relationship between CO_2_ assimilation, photosynthetic electron transport, and active O_2_ metabolism in leaves of maize in the field during periods of low temperature. Plant Physiol..

[37] Huang Y., Lin C., He F., Li Z., Guan Y., Hu Q., Hu J. (2017). Exogenous spermidine improves seed germination of sweet corn via involvement in phytohormone interactions, H_2_O_2_ and relevant gene expression. BMC Plant Biol..

[38] Li Z., Peng Y., Zhang X.-Q., Ma X., Huang L.-K., Yan Y.-H. (2014). Exogenous spermidine improves seed germination of white clover under water stress via involvement in starch metabolism, antioxidant defenses and relevant gene expression. Molecules.

[39] Bagni N. (1970). Metabolic changes of polyamines during the germination of *Phaseolus vulgaris*. New Phytol..

[40] Villanueva V.R., Adlakha R.C., Cantera-Soler A.M. (1978). Changes in polyamine concentration during seed germination. Phytochemistry.

[41] Liu Y.-Z., Tang B., Zheng Y.-L., Ma K.-J., Xu S.-Z., Qiu F.-Z. (2010). Screening methods for waterlogging tolerance at maize (*Zea mays* L.) seedling stage. Agr. Sci. China.

[42] Gill S.S., Tuteja N. (2010). Polyamines and abiotic stress tolerance in plants. Plant Signal. Behav..

[43] Nahar K., Hasanuzzaman M., Alam M.M., Rahman A., Mahmud J.-A., Suzuki T., Fujita M. (2017). Insights into spermine-induced combined high temperature and drought tolerance in mung bean: Osmoregulation and roles of antioxidant and glyoxalase system. Protoplasma.

[44] Blokhina O., Virolainen E., Fagerstedt K.V. (2003). Antioxidants, oxidative damage and oxygen deprivation stress: A review. Ann. Bot..

[45] Ozga J.A., Reinecke D.M., Knowles N.R., Blenis P. (2004). Characterization of the loss of seedling vigor in pea (*Pisum sativum* L.). Can. J. Plant Sci..

[46] Kranner I., Roach T., Beckett R.P., Whitaker C., Minibayeva F.V. (2010). Extracellular production of reactive oxygen species during seed germination and early seedling growth in *Pisum sativum*. J. Plant Physiol..

[47] Verma G., Mishra S., Sangwan N., Sharma S. (2015). Reactive oxygen species mediate axis-cotyledon signaling to induce reserve mobilization during germination and seedling establishment in *Vigna radiata*. J. Plant Physiol..

[48] Tamang B.G., Magliozzi J.O., Maroof M.A.S., Fukao T. (2014). Physiological and transcriptomic characterization of submergence and reoxygenation responses in soybean seedlings. Plant Cell Environ..

[49] Li Z., Zhou H., Peng Y., Zhang X., Ma X., Huang L., Yan Y. (2015). Exogenously applied spermidine improves drought tolerance in creeping bentgrass associated with changes in antioxidant defense, endogenous polyamines and phytohormones. Plant Growth Regul..

[50] Wang X., Shi G., Xu Q., Hu J. (2007). Exogenous polyamines enhance copper tolerance of *Nymphoides peltatum*. J. Plant Physiol..

[51] Nayyar H., Chander S. (2004). Protective effects of polyamines against oxidative stress induced by water and cold stress in chickpea. J. Agron. Crop Sci..

[52] Guerra D., Crosatti C., Khoshro H.H., Mastrangelo A.M., Mica E., Mazzucotelli E. (2015). Post-transcriptional and post-translational regulations of drought and heat response in plants: A spider’s web of mechanisms. Front. Plant Sci..

[53] Handa A.K., Mattoo A.K. (2010). Differential and functional interactions emphasize the multiple roles of polyamines in plants. Plant Physiol. Biochem..

[54] Wang C., Fan L., Gao H., Wu X., Li J., Lv G., Gong B. (2014). Polyamine biosynthesis and degradation are modulated by exogenous gamma-aminobutyric acid in root-zone hypoxia-stressed melon roots. Plant Physiol. Biochem..

[55] Wimalasekera R., Tebartz F., Scherer G.F.E. (2011). Polyamines, polyamine oxidases and nitric oxide in development, abiotic and biotic stresses. Plant Sci..

[56] Moschou P.N., Wu J., Cona A., Tavladoraki P., Angelini R., Roubelakis-Angelakis K.A. (2012). The polyamines and their catabolic products are significant players in the turnover of nitrogenous molecules in plants. J. Exp. Bot..

